# Continuous anti-angiogenic therapy after tumor progression in patients with recurrent high-grade epithelial ovarian cancer: phase I trial experience

**DOI:** 10.18632/oncotarget.9048

**Published:** 2016-04-27

**Authors:** Ming-Mo Hou, Zhijie Wang, Filip Janku, Sarina Piha-Paul, Aung Naing, David Hong, Shannon Westin, Robert L. Coleman, Anil K. Sood, Apostolia M. Tsimberidou, Vivek Subbiah, Jennifer Wheler, Ralph Zinner, Karen Lu, Funda Meric-Bernstam, Siqing Fu

**Affiliations:** ^1^ Departments of Investigational Cancer Therapeutics, The University of Texas MD Anderson Cancer Center, Houston, Texas, USA; ^2^ Departments of Gynecologic Oncology and Reproductive Medicine, The University of Texas MD Anderson Cancer Center, Houston, Texas, USA; ^3^ Division of Hematology-Oncology, Chang Gung Memorial Hospital and Chang Gung University, Taoyuan, Taiwan; ^4^ Department of Thoracic Medical Oncology, Key Laboratory of Carcinogenesis and Translational Research (Ministry of Education), Peking University Cancer Hospital & Beijing Institute for Cancer Research, Beijing, China

**Keywords:** epithelial ovarian cancer, anti-angiogenesis, tumor progression, progression-free survival, overall survival

## Abstract

High-grade epithelial ovarian cancer (HG-EOC) is the most lethal gynecologic malignancy worldwide Once patients develop chemoresistance, effective novel strategies are required to improve prognosis We analyzed characteristics and outcomes of 242 consecutive patients with HG-EOC participating in 94 phase I clinical trials at The University of Texas MD Anderson Cancer Center. Baseline lactate dehydrogenase levels, albumin levels, and number of metastatic sites were independent predictors of overall survival (OS). Receiving more than 1 phase I protocol was associated with improved OS (p < 0.001). Regimens including a chemotherapeutic agent plus bevacizumab or Aurora A kinase inhibitor led to a median progression-free survival (PFS) duration of more than 6 months. Although patients receiving bevacizumab-based regimens in the phase I clinical trials had significantly longer PFS than those receiving other anti-angiogenic therapies (p = 0.017), patients treated with vascular endothelial growth factor receptor-tyrosine kinase inhibitors (VEGFR-TKIs) had significantly longer OS (12.2 months) than those not treated with VEGFR-TKIs (8.6 months, p = 0.015).

In conclusion, anti-angiogenic therapy is one of the most important strategies for the treatment of HG-EOC, even in those who have already experienced tumor progression. Therefore, eligible patients with HG-EOC should be encouraged to participate in novel phase I studies of anti-angiogenic therapies, even after disease progression.

## INTRODUCTION

Epithelial ovarian cancer (EOC) is the most lethal gynecologic malignancy worldwide and the fourth most common cause of cancer-related death in women [1]. As a major histologic type, high-grade EOC (HG-EOC) constitutes more than 80% of ovarian cancer and most frequently presents as advanced-stage disease with poor survival prognosis [2, 3]. Cytoreductive surgery after neoadjuvant therapy or followed by platinum-based chemotherapy is a popular and effective management strategy [4, 5]. However, despite satisfactory clinical outcomes after initial therapy, most patients suffer relapse [6, 7]. Subsequent chemotherapy is only modestly effective, even for platinum-sensitive patients. Once patients develop chemoresistance, effective novel strategies are required to improve prognosis [6].

Although many new cytotoxic agents and therapeutic schedules are being developed, a therapeutic bottleneck appears to have been reached. The emergence of targeted therapeutic agents, based in part by an expanding understanding of the biological systems governing oncogenesis, brought promise to these patients [8]. The most extensively investigated targeted regimens have been anti-angiogenic agents, including the vascular endothelial growth factor (VEGF) antibody bevacizumab [9–12] and VEGF receptor-tyrosine kinase inhibitors (VEGFR-TKIs) [13–16]. Though a total of 8 phase III trials with these agents have been conducted in the frontline, maintenance and recurrent disease setting with uniform improvement in progression-free survival [6], bevacizumab is FDA-approved in combination with chemotherapy only for patients with platinum-resistant recurrent ovarian cancer [6]. Poly (adenosine diphosphate [ADP]-ribose) polymerase inhibitors have also been evaluated as part of single or combined therapy, and these demonstrated superior efficacy in EOC patients with germline BRCA1/2 (*gBRCA-mt*) mutations [17–19]. The FDA granted accelerated approval to olaparib, the PARP inhibitor, for the treatment of recurrent *gBRCA-mt* EOC patients who have been treated with 3 or more prior lines of therapy. Other promising targeted agents developed for the treatment of EOC include agents targeting PI3K-AKT-mTOR [20, 21], MAPK [22], Src [23, 24], Wee1 [25], and Aurora A kinase signaling pathways [26, 27].

Beyond investigations of safety and tolerance of newly developed targeted agents, phase I clinical trials provide the first step in the delivery of future potential therapeutic regimens. However, with many phase I clinical trials currently in progress and a growing number of patients with refractory disease, it is difficult to determine which treatments are most likely to lead to good outcomes and which patients would most benefit from enrolling in the trials. We here in report the clinical characteristics and outcomes of a large cohort of consecutive patients with advanced HG-EOC participating in phase I clinical trials at The University of Texas MD Anderson Cancer Center (MD Anderson), to identify the most promising potential novel regimens for the treatment of patients with HG-EOC.

## RESULTS

### Patient characteristics

In the cohort of 242 patients enrolled in phase I clinical trials, most patients were white (80%), had good ECOG performance status of 1 or better (96%), were diagnosed with serous carcinoma (79%), and had stage III-IV disease (90%) at their initial diagnosis (Table 1). In patients who had molecular profiling performed, the most frequent gene aberration was a *TP53* hotspot mutation (49 out of the 79 patients tested; 62%). Other molecular aberrations included BRCA1/2 (8/18, 44%), PIK3CA (17/190, 9%), KRAS (15/168, 9%), c-KIT (7/102, 7%), KDR (3/54, 6%), B-RAF (3/163, 2%), NRAS (2/111, 2%), AKT-1 (1/93, 1%), EGFR (1/149, 1%), and MET amplification (7/119, 6%). No ALK rearrangement (84 patients tested), GNAS mutations (66 patients tested), or GNAQ mutations (54 patients tested) were detected. Fluorescence *in situ* hybridization (FISH) analysis revealed Her-2 amplification (5/126, 4%), and immunohistochemistry showed complete PTEN loss (4/155, 3%) and estrogen receptor or progesterone receptor expression anomalies (114/242, 47%).

**Table 1:** Characteristics of the 242 patients enrolled in phase I clinical trials during the period studied

Characteristic	No. (%)
Median age (range)	64 years (27-79 years)
Race/ethnicity	
White	194 (80)
African-American	20 (8)
Hispanic	12 (5)
Asian	16 (7)
Pathologic diagnosis (all high-grade)	
Serous carcinoma	192 (79)
Clear cell carcinoma	24 (10)
Poorly differentiated/undifferentiated carcinoma	19 (8)
Endometrioid carcinoma	7 (3)
Initial disease stage	
I-II	23 (10)
III-IV	219 (90)
Eastern Cooperative Oncology Group performance status	
0	66 (27)
≥1	176 (73)
Lactate dehydrogenase	
Normal	143 (59)
Above normal	99 (41)
Albumin	
≥3.5 g/dL	214 (88)
<3.5 g/dL	28 (12)
Metastatic sites	
≤2	156 (64)
>2	86 (36)
Prior anti-angiogenic therapy	
Yes	123 (51)
No	119 (49)
Prior systemic therapy	
1 prior line	21 (9)
2 prior lines	30 (12)
≥3 prior lines	191 (79)
Median no. of prior therapies (range)	4 (1-16)
No. of phase I protocols enrolled	
1	161 (67)
≥2	81 (33)

### Disease response to phase I trial therapy

Two hundred forty-two patients received 358 types of therapy under 94 different phase I clinical trials conducted at MD Anderson during the period studied. Among these 242 patients, 160 received therapy as a participant in 1 phase I clinical trial, 48 from 2 phase I clinical trials, and 34 from 3 or more clinical trials. The 94 phase I clinical trials were classified as follows: single-agent targeted therapy (n = 33), combined targeted therapy (n = 31), targeted therapy plus chemotherapy (n = 22), chemotherapy alone (n = 7), and immunotherapy (n = 1). The 358 therapies were grouped as matched therapy (n = 59) or unmatched/known therapy (n = 299), and anti-angiogenic therapy (n = 224) or non-anti-angiogenic therapy (n = 134).

**Figure 1:**
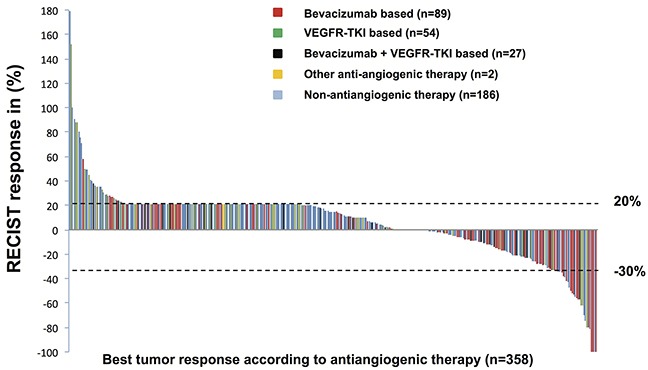
Waterfall plot shows the best objective responses according to Response Evaluation Criteria in Solid Tumors (RECIST) All 358 phase I clinical trial therapies administered to 242 patients are shown. A 21% or higher RECIST response represents new lesions, early tumor progression, or early withdrawal from treatment for other reasons; this may be arbitrarily designated 21% or higher disease progression or actual tumor progression of 21% or higher. VEGFR-TKI, vascular endothelial growth factor receptor-tyrosine kinase inhibitor.

The overall objective response rate across the 358 therapies in our analysis was 9% (complete response: n = 4, 1%; partial response: n = 30, 8%) and the stable disease rate was 45% (n = 162), as shown in Figure 1. The median duration of response was 8.6 months. No difference in objective response was observed between matched (9% objective response across 59 therapies) and unmatched/known therapy (10% objective response across 299 therapies; *p* = 0.77). Targeted therapy plus chemotherapy was associated with a significantly higher objective response (20/112, 18%) than other types of therapy (14/246, 6%; *p* < 0.001). The objective response rate observed in trials using a bevacizumab-based regimen (16/89, 18%), had a significantly better response rate than other regimens (*p* = 0.019): VEGFR-TKI-based regimens (4/54, 7%), bevacizumab plus VEGFR-TKIs (2/27, 7%), and non-anti-angiogenic therapies (12/188, 6%). No patients experiencing multiple lines of therapy on phase I trials of anti-angiogenic therapies had symptoms and/or radiographic findings of small bowel obstruction.

### PFS

**Figure 2:**
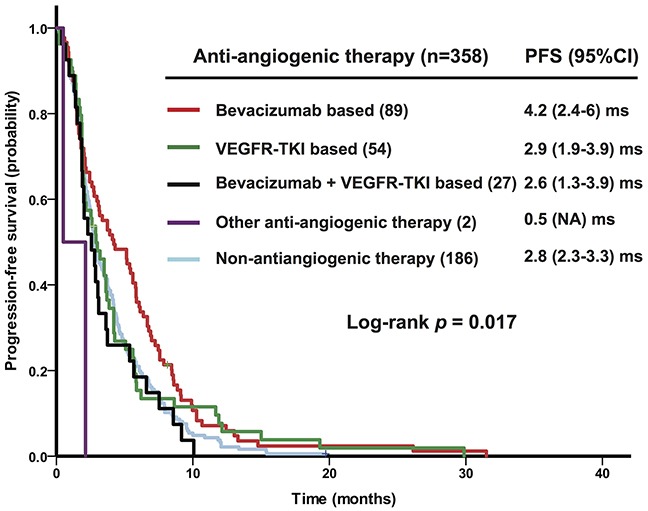
Kaplan-Meier plot shows progression-free survival (PFS) after phase I therapies (358 phase I clinical trial therapies) administered to 242 patients with recurrent high-grade epithelial ovarian cancer according to the type of therapy VEGFR-TKI, vascular endothelial growth factor receptor-tyrosine kinase inhibitor.

The median PFS of 358 phase I treats delivered in 242 consecutive patients with recurrent HG-EOC who received their phase I clinical trial therapy at our phase I trial clinic was 3 months (95% confidence interval [95%CI], 2.6-3.4 months). Univariate analyses demonstrated that the prolonged PFS after treatment in a phase I clinical trial was associated with normal albumin levels (*p* = 0.005), 2 or fewer metastatic sites (*p* = 0.049), and prior anti-angiogenic therapy (*p* = 0.028). Patients receiving bevacizumab-based regimens (n = 89 therapies) had significantly longer PFS (4.2 months) than those receiving other therapies (*p* = 0.017), including VEGFR-TKI-based regimens (n = 54 therapies, 2.9 months), bevacizumab plus VEGFR-TKIs (n = 27 therapies, 2.6 months), and non-anti-angiogenic therapy (n = 188 therapies, 2.8 months), as shown in Figure 2.

Patients who received targeted therapy combined with chemotherapy had a median PFS of 4.1 months, which compared favorably (*p* = 0.074) with other strategies, including single-agent targeted therapy (2.4 months), combination of targeted therapies (2.9 months), and chemotherapy alone (2.5 months). Preliminary data revealed that phase I clinical trial therapy with bevacizumab and nab-paclitaxel plus gemcitabine, Aurora A kinase inhibitor, or Aurora A kinase inhibitor plus paclitaxel led to a median PFS of greater than 6 months.

The multivariable Cox proportional hazards model, which included the factors of age (<60 years or ≥60 years), race/ethnicity (white or nonwhite), pathologic diagnosis (serous carcinoma or other), ECOG performance status (0 or ≥1), LDH (normal or above normal), albumin (≥3.5 g/dL [normal] or <3.5 g/dL), number of metastatic sites (≤2 or >2), prior anti-angiogenic therapy (yes or no), previous lines of systemic therapy (<3 or ≥3), first-time delivery of therapy in a phase I clinical trial (yes or no), and therapy match of the treatment in the phase I clinical trial (matched or unmatched/known), showed that age <60 years (*p* = 0.022), albumin ≥3.5 g/dL (*p* = 0.007), and no prior anti-angiogenic therapy (*p* = 0.007) were independent predictors of prolonged PFS.

### OS

In all 242 patients who received therapy in a phase I clinical trial, a median OS of 38.2 months from the initial clinic visit observed, which was similar to that of patients referred to our clinic who did not receive treatment in a phase I clinical trial (n = 83, 40.9 months; *p* = 0.12). Among the patients who received therapy, a median OS of 44.3 months from the date of initial recurrence was observed in patients who received subsequent therapies with bevacizumab and VEGFR-TKI-based therapies, which compared favorably, although not statistically significant (*p* = 0.64), with those who had only 1 line of anti-angiogenic therapy (36.4 months) or no anti-angiogenic therapy (32.7 months).

**Figure 3:**
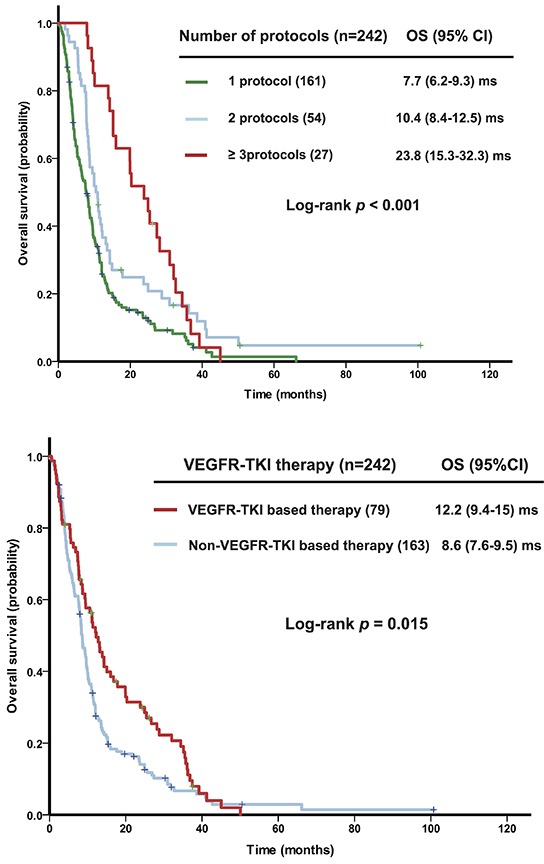
Kaplan-Meier plots shows overall survival (OS) in patients with recurrent high-grade epithelial ovarian cancer (n = 242) according to **A.** the number of therapies received in phase I clinical trials and **B.** the type of therapy received (vascular endothelial growth factor receptor-tyrosine kinase inhibitor [VEGFR-TKI]-based therapy or other).

Univariate analyses of factors affecting OS from initiation of the first therapy in a phase I clinical trial (n = 242 patients) revealed that normal LDH levels (*p* = 0.009), albumin ≥3.5 g/dL (*p* < 0.001), ≤2 metastatic sites (*p* < 0.001), and no prior anti-angiogenic therapy (*p* = 0.013) were associated with prolonged OS (Table 2). Patients receiving therapy in 3 or more phase I clinical trials had a median OS of 23.8 months, which was significantly better (*p* < 0.001) than those receiving therapy in 2 trials (10.4 months) or only 1 trial (7.7 months), as shown in Figure 3A. Patients who received VEGFR-TKI therapy (n = 79) had a median OS of 12.2 months, which was significantly longer than the median OS of 8.6 months in those who receive non-VEGFR-TKI therapy (n = 163, *p* = 0.015), as shown in Figure 3B.

**Table 2:** Univariate analyses of overall survival (OS) after treatment in a phase I clinical trial (n = 242 patients)

Factor	OS (95% confidence interval), months	*p*
Age		0.536
<60 years	9.4 (8.5-10.3)	
≥60 years	9.3 (7.0-11.6)	
Race/ethnicity		0.947
White	9.4 (8.2-10.6)	
Nonwhite	8.7 (6.8-10.6)	
Pathologic diagnosis		0.265
Serous carcinoma	9.4 (8.3-10.6)	
Other	9.2 (7.1-11.3)	
Eastern Cooperative Oncology Group performance status		0.165
0	10.1 (8.3-11.8)	
≥1	8.7 (7.7-9.7)	
Lactate dehydrogenase		0.009
Normal	11.1 (9.9-12.4)	
Above normal	8.1 (7.0-9.2)	
Albumin		<0.001
≥3.5 g/dL (normal)	10.1 (9.0-11.2)	
<3.5 g/dL	3.9 (3.0-4.7)	
Metastatic sites		<0.001
≤2	10.6 (8.9-12.3)	
>2	7.6 (5.2-9.9)	
Prior anti-angiogenic therapy		0.013
Yes	8.6 (7.1-10.1)	
No	11.0 (8.8-13.2)	
Prior systemic therapy		0.075
<3 lines	12.0 (8.4-15.7)	
≥3 lines	8.7 (7.8-9.6)	

The multivariable Cox proportional hazards model, which included the factors of age (<60 years or ≥60 years), race/ethnicity (white or nonwhite), pathologic diagnosis (serous carcinoma or other), ECOG performance status (0 or ≥1), LDH (normal or above normal), albumin (≥3.5 g/dL or <3.5 g/dL), number of metastatic sites (≤2 or >2), prior anti-angiogenic therapy (yes or no), and previous lines of systemic therapy (<3 or ≥3) showed that age <60 years (*p* = 0.029), normal LDH levels (*p* = 0.047), albumin ≥3.5 g/dL (*p* < 0.001), ≤2 metastatic sites (*p* < 0.001), and no prior anti-angiogenic therapy (p = 0.015) were independent factors predicting prolonged OS (Table 3).

**Table 3:** Multivariate analyses of overall survival after treatment in a phase I clinical trial (n = 242 patients)

Factor	*p*	Hazard ratio	95% confidence interval	
			Lower	Upper
Age <60 years	0.029	1.374	1.034	1.827
White	0.511	1.126	0.790	1.606
Serous carcinoma	0.109	1.324	0.939	1.866
ECOG performance status ≥1	0.310	1.174	0.861	1.599
Normal lactate dehydrogenase levels	0.047	1.342	1.004	1.795
Albumin ≥3.5 g/dL (normal)	<0.001	3.060	1.987	4.712
≤2 metastatic sites	<0.001	1.637	1.229	2.179
Prior antiangiogenic therapy	0.015	0.704	0.530	0.935
<3 prior lines of systemic therapy	0.644	1.091	0.755	1.576

## DISCUSSION

In the current study, we assessed a large cohort of patients with recurrent HG-EOC who were referred to the phase I clinical trials program at MD Anderson. Approximately 74% of the patients referred to the program during the period studied participated in a therapeutic phase I clinical trial. We found that anti-angiogenic therapy was one of the most important strategies for the treatment of HG-EOC. Chemotherapy plus bevacizumab-based or Aurora A kinase inhibitor-based regimens were potentially effective and yielded a median PFS of more than 6 months. Our preliminary evidence indicates that continuous anti-angiogenic therapy after tumor progression is associated with a significant survival benefit, and eligible patients should continue to participate in phase I clinical trials even if anti-angiogenic therapy from the first trial is not effective.

The tumor suppressor gene *TP53* is mutated in approximately 96% of cases of high-grade serous ovarian carcinoma [33]. *TP53* is at the hub of numerous signaling pathways involved in tumorigenesis, tumor development, and metastasis [34, 35], and it is triggered by a range of cellular stresses, such as transient cell cycle arrest, DNA repair, senescence, apoptosis, metabolism, stem cell maintenance, invasion, metastasis, and communication with the tumor microenvironment [36–38]. Mutations in *TP53* in cancer cells lead to accelerated tumor growth as a result of increased VEGF expression and neovascularization [39]. These processes represent an important survival pathway [40, 41], and VEGF inhibition in patients with p53-mutant malignancies is therefore an effective therapeutic strategy [12, 13]. Bevacizumab, a monoclonal anti-VEGF-A antibody, has been shown to improve PFS when combined with chemotherapy compared with chemotherapy alone in several phase III studies, such as the ICON7 [9, 11], OCEANS [10], AURELIA [8], GOG218 [12], and GOG213 [5] trials. Likewise, in our study, anti-angiogenic therapies showed remarkable antitumor activity in the phase I clinical trials and were associated with increased PFS, demonstrating the importance of p53-VEGF crosstalk in ovarian cancer tumorigenesis and development and the therapeutic advantage of VEGF inhibition in this cohort of patients.

In the current study, we sought to determine whether prior exposure to bevacizumab-based therapy potentially affected the antitumor activity of further treatment with bevacizumab-based chemotherapeutic regimens in phase I studies. No difference was observed in overall objective responses and PFS between patients with prior bevacizumab exposure who received bevacizumab-based chemotherapeutic regimens in phase I clinical studies and those without prior exposure. Furthermore, continuation of anti-angiogenic therapy in combination with various other therapeutic agents after the patient had experienced tumor progression led to a median OS of 12.2 months, which was significantly greater than the median OS in those who did not continue anti-angiogenic therapy (8.6 months). These results are consistent with those of previous studies showing that maintenance of anti-angiogenic therapy with sequential VEGF inhibition-based regimens after disease progression has a clinical benefit in patients with advanced malignancies [42, 43]. This could be explained by the following reasons, as well as others: 1) the pro-angiogenic property of p53-VEGF crosstalk is essential to ovarian cancer tumorigenesis, development, and resistance to cancer therapy; 2) bevacizumab targets a common pathway mediating resistance to multiple cancer therapeutic agents, and therefore continuous treatment with bevacizumab sensitizes the cancer cells to subsequent therapeutic regimens; 3) because VEGF suppresses the tumor immune microenvironment, prolonged treatment with bevacizumab improves tumor response, leading to better antitumor activity in subsequent therapies; or 4) no or little overlapping toxicity between bevacizumab and other cancer therapeutic agents allows treatment with bevacizumab to be continued in subsequent lines of therapy. Moreover, we found that the more phase I clinical trials that patients participated in, the longer their overall survival duration. This finding suggests that patients with metastatic or recurrent HG-EOC should be encouraged to participate in more phase I clinical trials whenever they are eligible. Another explanation might be that the patients who lived longer got more treatment.

Our current data revealed no difference in antitumor activity between matched and unmatched therapy. This finding may simply indicate that the presence of *TP53* or other mutations predominantly in HG-EOC dilutes the therapeutic advantage of matched therapy, or that the genomic alterations targeted by the matched therapy reported here are not genuine driver mutations. In addition to anti-angiogenic therapy, another therapeutic strategy for HG-EOC is to target the Aurora kinase family, which plays a critical role in the regulation of chromosomal segregation and cytokinesis during mitotic progression. Aurora A kinase overexpression has been strongly linked with poor clinical outcomes, providing a potential therapeutic target in HG-EOC. In our study, treatment with an Aurora A kinase inhibitor as a single agent or in combination with chemotherapy produced promising antitumor activity, warranting further exploration.

In considering the clinical relevance and importance of our findings, several limitations and nuances should be noted. First, as is always inherent to retrospective methodology, the selection bias of patient referral to our phase I clinical trials program may limit the generalizability of our findings. The rate of phase I clinical trial enrollment for patients with HG-EOC (74%) is significantly higher than the average patient enrollment rate in phase I trials at our institution (~55%), as we previously described [44]. Second, small sample sizes in the subgroup analyses limit the validity of these statistical assessments. Finally, the actual reasons for declining to enroll in a phase I trial were unknown or not identified in the electronic medical records of patients in the period studied. Therefore, conclusions from this retrospective study should be considered preliminary evidence for the purpose of hypothesis generation, and these findings require further validation in larger prospective studies to confirm the benefit of continuously use of anti-angiogenic therapy after tumor progression.

In summary, we retrospectively reviewed the records of 242 consecutive patients with HG-EOC who received therapy in a phase I clinical trial. To the best of our knowledge, this research represents the largest retrospective study in patients with metastatic or recurrent HG-EOC who were referred to a phase I clinical trial. Our study revealed the potential efficacy and importance of therapeutic strategies to target angiogenesis and Aurora A kinase, as single agents or in combination with chemotherapy, for the treatment of HG-EOC. Preliminary data indicated that continuous VEGF inhibition in combination with various other therapeutic agents was associated with improved clinical outcomes. Patients with advanced HG-EOC who are eligible should be encouraged to try novel therapeutic regimens in clinical trials.

## MATERIALS AND METHODS

### Patients

Three hundred twenty-five consecutive patients with histologically confirmed HG-EOC, including high-grade papillary serous, poorly differentiated, endometrioid, and clear cell carcinoma, were referred to the phase I clinical trials program at MD Anderson between May 1, 2006, and December 31, 2014. Among these patients, we reviewed the records of the 242 consecutive patients (74%) who had actually received treatment as a participant in at least 1 phase I clinical trial during the period studied. We obtained patient demographics, medical history, Eastern Cooperative Oncology Group (ECOG) performance status, laboratory results, gene aberration status, and status or outcome of treatment administered in the phase I clinical trial. Trial procedures, data collection, and the subsequent analysis were performed in accordance with the guidelines of the MD Anderson Institutional Review Board.

### Molecular analysis

Molecular profiling via next-generation sequencing has only recently become available. When adequate tissue samples were available for patients in the period studied, molecular analyses were performed. DNA was extracted from microdissected paraffin-embedded tumor specimens and gene aberrations were detected at the Clinical Laboratory Improvement Amendments-certified MD Anderson Molecular Diagnostics Laboratory [28, 29].

### Treatment and evaluation

The decision to enroll an eligible patient into a phase I clinical trial varied over time, depending on the protocol availability and the preference of the treating physician. The treatment in a phase I clinical trial was considered matched therapy if at least 1 drug in the regimen was known to inhibit the functional activity of at least 1 of the patient's gene aberrations. Toxic effects were assessed according to the National Cancer Institute Common Terminology Criteria for Adverse Events version 3.0 or 4.0 [30], and tumor response was evaluated according to the Response Evaluation Criteria in Solid Tumors version 1.0 or 1.1 [31, 32], depending on the individual protocol. The usual surveillance for tumor treatment effect was performed every other cycle (one every 6 or 8 weeks). Progression-free survival (PFS) was calculated from the date of initiation of treatment in a phase I clinical trial to the first objective documentation of disease progression, the date of death, or the last date censored as of September 30, 2015. Overall survival (OS) was calculated from the date of the date of initiation of treatment in a phase I clinical trial to the date of death or the last date censored as of September 30, 2015.

### Statistical analysis

Patient characteristics, including age, race/ethnicity, pathologic type, disease stage, ECOG performance status, lactate dehydrogenase (LDH) levels, albumin levels, number of metastatic sites, previous systemic treatment, prior anti-angiogenic therapy, therapy match of the treatment in the phase I clinical trial, number of phase I clinical trials in which the patient enrolled, and gene aberrations were summarized using frequency distributions and percentages. A waterfall plot analysis was used to illustrate antitumor efficacy according to the type of treatment delivered in the phase I clinical trial (anti-angiogenic or non-anti-angiogenic). Categorical variables were compared via chi-square and Fisher exact tests. Survival durations (PFS and OS) were assessed using Kaplan-Meier curves by the log-rank test. A multivariable Cox proportional hazards model was used for multivariate analysis. All tests were 2-sided and considered significant when the *p* value was less than 0.05. Statistical analyses were performed using SPSS version 23.0 software (SPSS, Chicago, IL).
